# Relation between serum magnesium and outcome in patients with *Escherichia coli* sepsis

**DOI:** 10.1186/s12879-025-10979-3

**Published:** 2025-04-28

**Authors:** Yan Cao, Bangqi Hu, Wei Zhou, Zhengyu Liu, Yanfang Pei, Jiang Yu, Conglong Hu, Xin Liu, Xiaotong Han, Xiquan Yan, Liudang He, Ning Ding

**Affiliations:** 1 Department of Emergency Medicine, Hunan Provincial People’s Hospital (The First Affiliated Hospital of Hunan Normal University), Changsha, China; 2 Sepsis Research Center of Hunan Provincial Geriatric Institute, Hunan Provincial People’s Hospital (The First Affiliated Hospital of Hunan Normal University), Changsha, China; 3 Department of Emergency Medicine, The First Affiliated Hospital of Hunan Normal University, Changsha, China; 4 Department of Cardiology, Hunan Provincial People’s Hospital (The First Affiliated Hospital of Hunan Normal University), Changsha, China; 5 Clinical Research Center for Heart Failure of Hunan Province, Hunan Provincial People’s Hospital (The First Affiliated Hospital of Hunan Normal University), Changsha, China; 6 Institute of Emergency Medicine, Hunan Provincial Key Laboratory of Emergency and Critical Care Metabonomics, Hunan Provincial People’s Hospital (The First Affiliated Hospital of Hunan Normal University), Changsha, China; 7 Department of Emergency Medicine, Hengyang Medical School, The Affiliated Changsha Central Hospital, University of South China, NO.161 Shaoshan South Road, Changsha, 410004 Hunan China

**Keywords:** *E. coli* sepsis, Serum magnesium, Mortality, MIMIC-IV database

## Abstract

**Objective:**

*Escherichia coli* (*E.coli*) *is* the leading pathogen for deaths associated with antimicrobial resistance, making it the most problematic bacteria for human infections. This study aimed to investigate the association between serum magnesium levels and clinical outcomes in patients with *E.coli* sepsis.

**Method:**

Data of *E.coli* septic patients were collected from the MIMIC-IV database. Patients were divided into three groups based on tertiles of serum magnesium levels. Three models were utilized, including the raw model (unadjusted), Model I (adjusted for age and gender), and Model II (adjusted for all potential confounding factors). Linear model and two-segment nonlinear model were established to examine the relationship between serum magnesium and 30-day, 60-day, and 90-day mortality rates. Kaplan-Meier survival curve analysis was performed to assess cumulative hazard of mortalities at 30-day, 60-day, 90-day based on tertiles of serum magnesium levels.

**Results:**

A total of 421 *E.coli* septic patients were included and classified into tertiles: Q1(< 1.6 mg/dL), Q2 (1.6-1.9 mg/dL), Q3(> 1.9 mg/dL). In the Model adjusting for all potential confounders, for every 1 mg/dL increase in serum magnesium, there was a significant increase in 30-day, 60-day, and 90-day mortality rates, with odds ratios of 4.01 (95% CI 1.22–13.19, *P* = 0.022), 4.81 (95% CI 1.59–14.53, *P* = 0.005), and 4.45 (95% CI 1.52–12.96, *P* = 0.006) respectively. And linear model is more suitable for describing the relationship between serum magnesium levels and clinical outcomes. Kaplan-Meier analysis revealed that the cumulative hazard of mortalities at 30-day, 60-day, 90-day increased with the prolongation of hospital stay, particularly in the group with the highest serum magnesium level.

**Conclusion:**

Increased level of serum magnesium is significantly associated with increased risk of 30-day, 60-day and 90-day mortality in a population of septic patients with *E.coli* infection.

## Introduction

Despite ever-increasing improvements in the diagnosis and management of sepsis, this condition is still an important cause of morbidity and mortality in hospitals [1]. A systematic review revealed that *Escherichia coli* (*E.coli*), a *gram*-negative bacillus, accounts for more than 25% of sepsis cases [2]. *E.coli* sepsis affects a number of patients, especially those with hematological malignancies, trauma, burns, solid cancers, etc. Robust evidence indicates that *E.coli* bacteremia releases a large amount of endotoxins, which activate the complement system. Uncontrolled complement activation leads to coagulopathy and thrombosis-induced inflammation, resulting in multiple organ failure or even death [3]. *E.coli* was the most frequently identified pathogen, mainly causing intra-abdominal-infections and skin and soft-tissue infections [4]. The incidence of antimicrobial resistance caused by *E. coli* is constantly increasing, making it the first human bacterial infection due to antibiotic-resistant mortality [5–7]. Thus, *E.coli* sepsis poses a significant global cost to human health.

Magnesium, the second most abundant intracellular cation, is essential for supporting and sustaining human health. Magnesium is involved in energy metabolism and protein synthesis and plays a critical role in more than 600 enzymatic reactions [8]. It is crucial for the function of all cells, especially excitable cells such as cardiomyocytes, neurons or skeletal muscles. In inflammatory diseases or conditions, it is generally thought that magnesium regulate inflammatory response by reducing oxidative stress and inhibiting proinflammatory molecules [9]. Magnesium has also been recognized as a crucial factor in the modulation of innate and adaptive immune systems [10]. Disruption of the normal immune response exacerbates the dysregulated inflammatory state in sepsis, resulting in a worse prognosis. Magnesium abnormalities are common in critically ill patients. A study based on a pediatric intensive care database reports that after controlling confounding factors, hypermagnesaemia is a risk factor to inpatient mortality in children with sepsis. It demonstrates that inpatient mortality is higher when serum magnesium levels reaches 1.0 mmol/L [11]. However, it follows that different studies have reached inconsistent conclusions. A single-centre observational study involved 150 septic patients reports that mortality rate was higher in the hypomagnesemia group when compared to the normal magnesemia group [12]. Magnesium, as the primary divalent cation, also plays a crucial role in the outer membrane structure of *E.coli*. It binds to the lipopolysaccharide layer of the outer membrane, contributing to the maintenance of its integrity and stability, which is essential for the survival and normal physiological functions of *E.coli* [13]. The absence of magnesium may lead to damage to the outer membrane structure, thereby affecting the viability of *E.coli*. Nevertheless, the correlation between serum magnesium and clinical outcomes in patients with *E.coli* sepsis has not been reported before. Only a few studies have been conducted on magnesium and animal models of *E.coli* sepsis. In a mouse model of sepsis induced by *E.coli* lipopolysaccharide, magnesium supplement blocks calcium influx by inhibiting the adenosine triphosphate gated calcium channel P2X purinoceptor, and then suppressing the function of Gasdermin D N-terminal and inhibiting pyroptosis [14].

Since *E.coli* is now one of the predominant organisms responsible for sepsis, and the predictive value of serum magnesium in *E.coli* sepsis needs to be investigated. Therefore, our study aimed to explore the prognostic value of serum magnesium for predicting clinical outcomes in patients with *E.coli* sepsis.

## Methods

### Database

The data used in this study were collected from the US public database Medical Information Mart for Intensive Care IV (MIMIC-IV) (https://mimic.mit.edu/iv/). The database records clinical data on patients treated at the intensive care unit of Bath Israel Deaconess Medical Center in Boston over a period of nearly 12 years, beginning in 2008 [15].

### Study design

According to the sepsis guideline version 3.0, the inclusion criteria were the presence of infection and a SOFA score ≥ 2 [16]. The exclusion criteria were as follows: lack of serum magnesium data during the 24 h after admission, more than 5% missing information for individual variables [17], and age under 18 years.

### Information and variables

Patients with sepsis who met the criteria were identified and analysed through the MIMIC-IV database. The following variables were extracted: age; sex; comorbidities, including hypertension, diabetes, coronary artery disease (CAD), and renal disease; vital signs, including heart rate (HR), diastolic blood pressure (DBP), systolic blood pressure (SBP), and respiratory rate (RR); laboratory findings, including serum magnesium, total calcium, alanine aminotransferase (ALT), aspartate aminotransferase (AST), international normalized ratio (INR), prothrombin time (PT), partial thromboplastin time (PTT), creatinine, urea nitrogen, haemoglobin, red blood cell (RBC), white blood cell (WBC), platelet (PLT), anion gap (AG), bicarbonate, lactate, and glucose; scoring systems, including sequential organ failure assessment (SOFA) and acute physiology and chronic health evaluation (APACHE II); and clinical outcomes, including intensive care unit (ICU) and hospital length of stay (LOS), and prognosis (mortality rates at 30, 60 and 90 days). The variables were collected within 24 h after admission to the hospital, and the initial data for each variable were used. The primary outcome was 30-day mortality and the secondary outcomes were 60-day and 90-day mortality.

### Statistical analysis

The data were statistically analysed via EmpowerStats (http://www.empowerstats.com) and the software package R. The results were deemed statistically significant if the *P* value was less than 0.05. Patients diagnosed with sepsis were divided into three groups based on their serum magnesium levels, which were classified into tertiles: Q1, serum magnesium < 1.6 mg/dL, *n* = 133; Q2, serum magnesium 1.6–1.9 mg/dL, *n* = 120; and Q3, > 1.9 mg/dL, *n* = 168 (Table 1). Differences in variables among the three groups were analysed as follows: the Mann‒Whitney U test was used for comparing continuous variables, whereas the chi‒square test was used for comparing categorical variables.As we have mentioned before, samples with missing data more than 5% for individual variable are excluded. But for those missing data no more than 5%, multiple imputation was used to estimate missing values for each variable [18–19]. Three different clinical outcomes, mortality rates at 30, 60 and 90 days, were compared via univariate analysis. The relationship between serum magnesium and the different clinical outcomes was explored by three models. Crude model included only the serum magnesium with no adjustment. Model I was adjusted for age and gender. Model II, however, is a fully adjusted model, considering both feature selection outcomes and clinical expertise-based adjustments [20], confounders included age; gender; hypertension, diabetes, CAD, renal disease; HR, DBP, SBP, RR; total calcium, ALT, AST, total bilirubin, INR, PT, PTT, creatinine, urea nitrogen, hematocrit, hemoglobin, RBC, WBC, PLT, AG, lactate, glucose; APACHEII, SOFA. Furthermore, we employed two models (linear Model I and two-segment nonlinear model II) for comparison. According to the results of the log-likelihood ratio, results greater than 0.05 indicate a linear correlation, and results less than 0.05 indicate a nonlinear correlation. Finally, Kaplan‒Meier analyses of survival probabilities were constructed for the three groups (Q1‒Q3).

**Table 1:** Sepsis cohort description and variable comparison

Serum magnesium(mg/dL)					
Variables	Total	Q1(< 1.6)	Q2(1.6–1.9)	Q3(> 1.9)	*P*-value
Number	421	133	120	168	
Age(years)(median, IQR)	70.00 (60.00–81.00)	66.00 (57.00–77.00)	75.00 (64.00–84.00)	68.50 (60.00–81.00)	0.007
**Gender(*n***, **%)**					0.675
Male	200 (47.51%)	67 (50.38%)	57 (47.50%)	76 (45.24%)	
Female	221 (52.49%)	66 (49.62%)	63 (52.50%)	92 (54.76%)	
**Comorbidities(*n***, **%)**					
Hypertension	196 (46.56%)	63 (47.37%)	58 (48.33%)	75 (44.64%)	0.805
Diabetes	24 (5.70%)	10 (7.52%)	6 (5.00%)	8 (4.76%)	0.548
CAD	89 (21.19%)	34 (25.56%)	24 (20.00%)	31 (18.56%)	0.314
Renal disease	17 (4.05%)	5 (3.76%)	5 (4.17%)	7 (4.19%)	0.979
**Vital signs (median**, ** IQR)**					
HR(beats/min)	98.00 (83.00-110.00)	103.00 (88.00-115.00)	92.00 (80.00-104.00)	99.00 (81.75-110.25)	0.001
DBP(mmHg)	60.00 (51.00–71.00)	57.00 (50.00–67.00)	60.00 (50.00–70.00)	62.00 (53.00–74.00)	0.261
SBP(mmHg)	108.00 (95.00-125.00)	103.00 (92.00-116.00)	110.50 (97.00-125.25)	112.00 (97.00-130.25)	0.004
RR(beats/min)	20.00 (17.00–25.00)	21.00 (17.00–25.00)	20.00 (17.00–24.00)	20.00 (16.00–25.00)	0.788
**Laboratory findings (median**, ** IQR)**					
Serum magnesium(mg/dL)	1.70 (1.50-2.00)	1.40(1.30–1.50)	1.70(1.60–1.80)	2.10(2.00-2.30)	< 0.001
ALT(IU/L)	40.00 (19.00-101.00)	45.00 (22.00-100.00)	30.50 (17.00-98.75)	40.00 (20.00-109.00)	0.342
AST(IU/L)	54.00 (29.00-118.00)	57.00 (33.00-126.00)	46.50 (26.00-103.25)	54.00 (32.00-125.00)	0.355
AG(mmol/l)	15.00 (13.00–18.00)	16.00 (13.00–19.00)	15.00 (13.00–17.00)	54.00 (32.00-125.00)	0.355
Bicarbonate(mmol/l)	21.00 (18.00–24.00)	20.00 (17.00–23.00)	21.00 (19.00–25.00)	22.00 (18.00-25.25)	< 0.001
Lactate(mmol/l)	2.10 (1.37-3.00)	2.10 (1.40-3.00)	1.90 (1.30–2.90)	2.15 (1.40–3.02)	0.383
Total bilirubin(mg/dl)	0.90 (0.50–2.30)	1.00 (0.60–2.30)	0.90 (0.45–1.90)	0.80 (0.40–2.80)	0.124
Total calcium (mg/dl)	7.90 (7.30–8.50)	7.40 (6.80–7.80)	8.00 (7.50–8.50)	8.20 (7.65–8.70)	< 0.001
Creatinine(mg/dl)	1.30 (1.00-1.90)	1.30 (1.00-1.90)	1.20 (0.90–1.80)	1.35 (1.00-2.10)	0.106
Urea nitrogen(mg/dl)	27.00 (18.00–40.00)	25.00 (17.00–34.00)	24.00 (18.00-33.25)	31.50 (20.00-50.25)	< 0.001
Hematocrit(%)	32.05 (28.10–36.20)	31.90 (27.90–35.50)	32.45 (27.98–37.02)	32.20 (28.20–35.60)	0.766
Hemoglobin(g/dl)	10.50 (9.10–12.00)	10.60 (9.00-11.70)	10.85 (9.10–12.30)	10.40 (9.30–11.70)	0.693
INR	1.40 (1.20–1.70)	1.45 (1.30–1.80)	1.40 (1.20–1.67)	1.30 (1.20–1.70)	0.999
PT(s)	15.10 (13.70–18.60)	15.75 (14.10-18.92)	15.05 (13.72–17.90)	14.70 (13.33–18.17)	0.907
APTT(s)	34.30 (29.60–40.80)	34.40 (30.20-39.95)	34.45 (29.57–42.92)	33.60 (29.20-40.35)	0.659
RBC(*10^12^/l)	3.52 (3.05–3.98)	3.53 (3.02–3.97)	3.52 (3.04-4.00)	3.48 (3.08–3.96)	0.977
WBC(*10^9^/l)	12.60 (7.75–19.20)	12.90 (6.95–19.80)	10.80 (7.50–16.80)	14.00 (9.00-20.40)	0.136
PLT(*10^9^/l)	161.50 (115.00-236.25)	148.00 (107.00-202.00)	176.00 (113.50-223.25)	175.00 (122.50-262.50)	0.001
Glucose(mg/dl)	121.00 (98.00-163.00)	124.00 (95.00-165.00)	121.00 (100.00-153.50)	120.00 (98.00-164.00)	0.199
**Scoring systems(median**, ** IQR)**					
APACHEII	12.00 (9.00–14.00)	12.00 (10.00–14.00)	11.00 (9.00–14.00)	12.00 (9.00–15.00)	0.207
SOFA	3.00 (2.00–4.00)	3.00 (2.00–5.00)	2.50 (2.00–4.00)	3.00 (2.00–4.00)	0.103
**Clinical outcomes**					
LOS of ICU(days)	2.88 (1.62–5.91)	2.79 (1.58–4.79)	2.90 (1.85–5.97)	2.91 (1.57–6.56)	0.764
LOS of hospital(days)	7.86 (5.41–13.59)	7.93 (5.45–11.91)	7.88 (5.38–12.80)	7.73 (5.41–14.96)	0.943
30-day mortality(n,%)	45 (10.69%)	11 (8.27%)	10 (8.33%)	24 (14.29%)	0.150
60-day mortality(n,%)	52 (12.35%)	12 (9.02%)	11 (9.17%)	29 (17.26%)	0.044
90-day mortality(n,%)	58 (13.78%)	15 (11.28%)	12 (10.00%)	31 (18.45%)	0.073

Comparison of different variables among three groups. CAD, coronary artery disease; HR, heart rate; SBP, systolic blood pressure; DBP, diastolic blood pressure; RR, respiratory rate; ALT, Alanine aminotransferase; AST, aspartate aminotransferase; AG, anion gap; INR, international normalized ratio; PT, prothrombin time; APTT, activated partial thromboplastin time; WBC, white blood cells; PLT, platelet; RBC, red blood cells; SOFA, sequential organ failure assessment; APACHE, acute physiology and chronic health evaluation; LOS, length of stay; ICU, intensive care unit; IQR, interquartile ranges

### Ethical approval

The study adhered to the guidelines of the Declaration of Helsinki (2002) and utilized the anonymized MIMIC-IV public database. To access the database, the Protection of Human Research Participants Examination (No. 32900964) was passed, and approval and informed consent waivers were obtained from the Institutional Review Boards of Massachusetts Institute of Technology (MIT) and Beth Israel Deaconess Medical Center (BIDMC).

## Results

### Sepsis cohort description and variable comparison

**Fig. 1:**
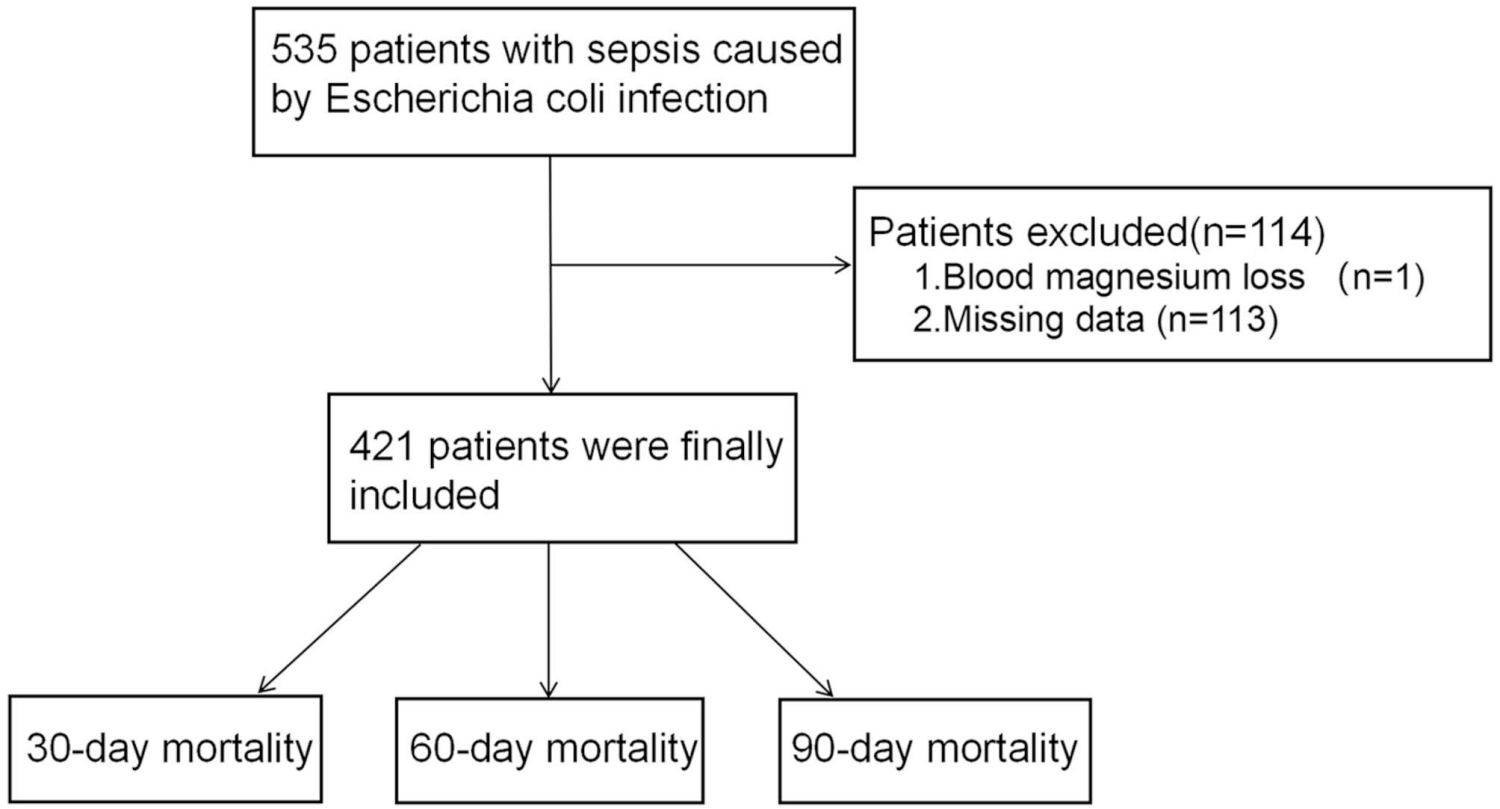
Flowchart demonstrating the selection of participants from the MIMIC-IV database

Initially, the inclusion and exclusion criteria led to the enrolment of 535 septic patients with *E.coli* infection in this study (Fig. 1: flowchart). Among them, one patient had serum magnesium loss, and 113 patients lost more than 5% of their data. Eventually, 421 patients who met the inclusion criteria were included in the analysis. The different baseline characteristics are illustrated in Table 1. The mean age of all the participants was 70 years; males comprised 47.51% (*n* = 200) of the sample, while females comprised 52.49% (*n* = 221) of the sample. In septic patients with *E.coli*, electrolyte imbalance is commonly observed. The laboratory analysis revealed that the total calcium content was 7.90 mg/dL, which is greater than the normal limit. The average bicarbonate concentration was 21 mmol/L, which is below the normal range.

We did a comparison of the variables among the three groups based on tertiles of serum magnesium levels. There were notable differences in terms of age (*P* = 0.007), HR (*P* = 0.001), SBP (*P* = 0.004), bicarbonate (*P* < 0.001), total calcium (*P* < 0.001), serum magnesium (*P* < 0.001), urea nitrogen (*P* < 0.001), and platelet count (*P* = 0.001). Serum magnesium, bicarbonate, total calcium, urea nitrogen and platelets in the Q3 group were obviously elevated compared to those in the Q1-Q2 groups. The APACHE II and SOFA scores did not significantly differ among the three groups.

For the variables related to clinical outcomes, the median length of stay (LOS) in the intensive care unit (ICU) was 2.88 days, whereas the median LOS in the hospital was 7.86 days. The 30-day, 60-day and 90-day mortality rates were 10.69% (*n* = 45), 12.35% (*n* = 52), and 13.78% (*n* = 58), respectively. The 60-day mortality rate in the Q3 group was significantly greater than that in the Q1-Q2 groups (*P* = 0.044).

### Univariate analysis of different outcomes in septic patients

In Table 2, univariate analysis was performed for 30-day, 60-day, and 90-day mortality. Four variables, namely, the lactate level, serum magnesium concentration, APACHE II score and SOFA score, were strongly associated with 30-day, 60-day, and 90-day mortality. Notably, for each 1 mg/dL increase in serum magnesium, the odds ratios (ORs) for 30-day, 60-day, and 90-day mortality were 2.64 (95% CI 1.33–5.25, *P* = 0.006), 2.69 (95% CI 1.40–5.16, *P* = 0.003), and 2.25 (95% CI 1.20–4.21, *P* = 0.011), respectively. For lactate level, ORs for 30-day, 60-day, and 90-day mortality were 1.33 (95% CI 1.13–1.57, *P =* 0.001), 1.34 (95% CI 1.14–1.57, *P* < 0.001), and 1.32 (95% CI 1.13–1.54, *P* < 0.001), respectively. For the APACHE II score, the ORs for 30-day, 60-day, and 90-day mortality were 1.11 (95% CI 1.03–1.19, *P* = 0.008), 1.11 (95% CI 1.04–1.19, *P* = 0.004), and 1.11 (95% CI 1.04–1.19, *P* = 0.002), respectively. For the SOFA score, the ORs for 30-day, 60-day, and 90-day mortality were 1.18 (95% CI 1.02–1.38, *P* = 0.029), 1.20 (95% CI 1.04–1.39, *P* = 0.012), and 1.18 (95% CI 1.03–1.36, *P* = 0.018), respectively.

**Table 2:** Univariate analysis for different outcomes in sepsis

Variables	Univariable (OR, 95%CI, *P*-value) (30-day mortality)	Univariable (OR, 95%CI, *P*-value) (60-day mortality)	Univariable (OR, 95%CI, *P*-value) (90-day mortality)
Age(years)	1.01 (0.99, 1.04) 0.201	1.01 (0.99, 1.03) 0.391	1.01 (0.99, 1.03) 0.439
Gender			
Male	Ref.	Ref.	Ref.
Female	0.70 (0.37, 1.30) 0.254	0.89 (0.50, 1.59) 0.701	1.05 (0.60, 1.82) 0.876
Hypertension			
No	Ref.	Ref.	Ref.
Yes	0.74 (0.40, 1.39) 0.352	0.75 (0.42, 1.36) 0.342	0.66 (0.38, 1.17) 0.158
CAD			
No	Ref.	Ref.	Ref.
Yes	0.81 (0.36, 1.81) 0.606	0.77 (0.36, 1.66) 0.510	0.87 (0.43, 1.77) 0.707
Diabetes			
No	Ref.	Ref.	Ref.
Yes	0.35 (0.05, 2.65) 0.308	0.63 (0.14, 2.76) 0.541	0.55 (0.13, 2.42) 0.432
Renal disease			
No	Ref.	Ref.	Ref.
Yes	2.79 (0.87, 8.97) 0.085	2.33 (0.73, 7.44) 0.153	2.81 (0.95, 8.31) 0.061
HR(beats/min)	1.01 (1.00, 1.03) 0.086	1.01 (1.00, 1.03) 0.069	1.01 (1.00, 1.02) 0.175
DBP(mmHg)	1.00 (0.98, 1.01) 0.789	0.99 (0.98, 1.01) 0.381	1.00 (0.98, 1.01) 0.596
SBP(mmHg)	1.00 (0.99, 1.01) 0.974	1.00 (0.99, 1.01) 0.975	1.00 (0.99, 1.01) 0.776
RR(beats/min)	1.03 (0.98, 1.08) 0.318	1.01 (0.97, 1.06) 0.556	1.00 (0.96, 1.05) 0.967
Serum magnesium(mg/dL)	2.64 (1.33, 5.25) 0.006	2.69 (1.40, 5.16) 0.003	2.25 (1.20, 4.21) 0.011
ALT(IU/L)	1.00 (1.00, 1.00) 0.640	1.00 (1.00, 1.00) 0.137	1.00 (1.00, 1.00) 0.177
AG(mmol/l)	1.03 (0.96, 1.10) 0.421	1.03 (0.96, 1.10) 0.410	1.03 (0.97, 1.10) 0.377
Lactate(mmol/l)	1.33 (1.13, 1.57) 0.001	1.34 (1.14, 1.57) < 0.001	1.32 (1.13, 1.54) < 0.001
AST(IU/L)	1.00 (1.00, 1.00) 0.632	1.00 (1.00, 1.00) 0.057	1.00 (1.00, 1.00) 0.077
Total bilirubin(mg/dl)	1.02 (0.93, 1.11) 0.717	1.02 (0.95, 1.11) 0.537	1.02 (0.95, 1.10) 0.510
Calcium total(mg/dl)	1.29 (0.98, 1.70) 0.068	1.16 (0.89, 1.52) 0.274	1.09 (0.83, 1.41) 0.541
Glucose(mg/dl)	1.00 (0.99, 1.00) 0.494	1.00 (0.99, 1.00) 0.292	1.00 (1.00, 1.00) 0.639
Creatinine(mg/dl)	1.11 (0.94, 1.31) 0.226	1.08 (0.92, 1.27) 0.364	1.08 (0.92, 1.26) 0.336
Urea nitrogen(mg/dl)	1.01 (1.00, 1.02) 0.195	1.00 (0.99, 1.01) 0.429	1.00 (1.00, 1.01) 0.306
Hematocrit(%)	1.01 (0.96, 1.06) 0.760	0.99 (0.94, 1.04) 0.709	0.99 (0.94, 1.03) 0.552
Hemoglobin(g/dl)	0.97 (0.83, 1.13) 0.708	0.93 (0.80, 1.07) 0.303	0.90 (0.78, 1.04) 0.156
INR	0.92 (0.66, 1.28) 0.619	0.89 (0.64, 1.25) 0.503	0.97 (0.76, 1.25) 0.833
PT(s)	0.99 (0.96, 1.02) 0.572	0.99 (0.96, 1.02) 0.465	1.00 (0.97, 1.02) 0.772
APTT(s)	1.00 (0.98, 1.02) 0.925	1.00 (0.99, 1.02) 0.951	1.00 (0.99, 1.01) 0.996
RBC(*10^12^/l)	1.02 (0.65, 1.59) 0.939	0.84 (0.55, 1.29) 0.422	0.82 (0.54, 1.23) 0.329
WBC(*10^9^/l)	1.00 (0.98, 1.03) 0.699	1.00 (0.98, 1.03) 0.779	1.01 (0.99, 1.03) 0.432
PLT(*10^9^/l)	0.99 (0.96, 1.02) 0.572	1.00 (1.00, 1.00) 0.979	1.00 (1.00, 1.00) 0.639
APAHCEII	1.11 (1.03, 1.19) 0.008	1.11 (1.04, 1.19) 0.004	1.11 (1.04, 1.19) 0.002
SOFA	1.18 (1.02, 1.38) 0.029	1.20 (1.04, 1.39) 0.012	1.18 (1.03, 1.36) 0.018

Univariate analysis was performed for 30-day, 60-day, 90-day mortality. OR, odds ratio; CI, confidential interval

### Correlation between the serum magnesium concentration and mortality according to the three models

Table 3 illustrates the associations of serum magnesium with three different outcomes, namely, 30-day mortality, 60-day mortality, and 90-day mortality. For the crude model (which included all the variables), every 1 mg/dL increase in serum magnesium led to an obvious increase in mortality rates at 30 days, 60 days, and 90 days, with OR values of 2.64 (95% CI 1.33–5.25, *P* = 0.006), 2.69 (95% CI 1.40–5.16, *P* = 0.003), and 2.25 (95% CI 1.20–4.21, *P* = 0.011), respectively. For Model I (with age and sex adjustments), every 1 mg/dL increase in serum magnesium led to a significant increase in the 30-day, 60-day, and 90-day mortality rates, with ORs of 2.71 (95% CI 1.35, 5.44, *P* = 0.005), 2.70 (95% CI 1.40, 5.20, *P* = 0.003), and 2.24 (95% CI 1.19–4.20, *P* = 0.012), respectively. For Model II (adjusted for all potential confounders), every 1 mg/dL increase in serum magnesium led to an obvious increase in mortality rates at 30 days, 60 days, and 90 days, with ORs of 4.01 (95% CI 1.22–13.19, *P* = 0.022), 4.81 (95% CI 1.59–14.53, *P* = 0.005), and 4.45 (95% CI 1.52–12.96, *P* = 0.006), respectively. The results indicated that every 1 mg/dL increase in serum magnesium led to obvious increases in 30-day, 60-day, and 90-day mortality rates of 301%, 381%, and 345%, respectively.

**Table 3:** Correlation between serum magnesium and mortality in different models

Exposure	Crude model	Model I	Model II
	OR, 95% CI, *P*-value	OR,95% CI, *P*-value	OR, 95% CI, *P*-value
**30-day mortality**			
Serum magnesium(per 1 mg/dL)	2.64 (1.33, 5.25) 0.006	2.71 (1.35, 5.44) 0.005	4.01 (1.22, 13.19) 0.022
Serum magnesium(mg/dL) tertile			
Q1	Ref.	Ref.	Ref.
Q2	1.01 (0.41, 2.47) 0.986	0.93 (0.38, 2.30) 0.872	2.93 (0.65, 13.20) 0.162
Q3	1.85 (0.87, 3.93) 0.110	1.84 (0.86, 3.93) 0.115	4.83 (1.19, 19.52) 0.027
*P* for trend	0.085	0.083	0.026
**60-day mortality**			
Serum magnesium(mg/dL)	2.69 (1.40, 5.16) 0.003	2.70 (1.40, 5.20) 0.003	4.81 (1.59, 14.53) 0.005
Serum magnesium(mg/dL) tertile			
Q1	Ref.	Ref.	Ref.
Q2	1.02 (0.43, 2.40) 0.968	0.96 (0.41, 2.30) 0.935	2.53 (0.64, 10.01) 0.186
Q3	2.10 (1.03, 4.30) 0.042	2.08 (1.01, 4.27) 0.046	5.87 (1.65, 20.88) 0.006
*P* for trend	0.028	0.028	0.005
**90-day mortality**			
Serum magnesium(mg/dL)	2.25 (1.20, 4.21) 0.011	2.24 (1.19, 4.20) 0.012	4.45 (1.52, 12.96) 0.006
Serum magnesium(mg/dL) tertile			
Q1	Ref.	Ref.	Ref.
Q2	0.87 (0.39, 1.95) 0.742	0.83 (0.37, 1.87) 0.654	2.50 (0.69, 8.99) 0.161
Q3	1.78 (0.92, 3.46) 0.089	1.75 (0.90, 3.40) 0.101	5.19 (1.58, 17.08) 0.007
*P* for trend	0.062	0.067	0.006

Correlation between serum magnesium and three different outcomes involving 30-day mortality, 60-day mortality, and 90-day mortality in three models. Serum magnesium was analyzed as continuous variable or categorical variable(Q1-Q3). Crude model: included all the variables; model I: adjusted for age and gender; model II: adjusted for age, gender, hypertension, diabetes, CAD, renal disease, HR, DBP, SBP, RR, calcium total, ALT, AST, BILIRUBINTOTAL, INR(s), PT(s), APTT(s), creatinine, Urea nitrogen, hematocrit, hemoglobin, RBC, WBC, PLT, AG, lactate, glucose, APACHEII, SOFA. OR, odds ratio; CI, confidential interval

To provide a more intuitive data analysis, we transformed the serum magnesium concentration as a continuous variable into a categorical variable (Q1-Q3). Compared with that in the Q1 group, the risk of 60-day mortality in the Q3 group was greater according to all three models, with ORs of 2.10 (95% CI 1.03, 4.30, *P* = 0.042), 2.08 (95% CI 1.01, 4.27, *P* = 0.046), 5.87 (95% CI 1.65, 20.88, *P* = 0.006), respectively). In Model II, the Q3 group had a greater risk of 30-day, 60-day, and 90-day mortality than the Q1 group, with ORs of 4.83 (95%CI 1.19–19.52, *P* = 0.027), 5.87 (95% CI 1.65–20.88, *P* = 0.006), and 5.19 (95% CI 1.58–17.08, *P* = 0.007), respectively.

### A linear relationship between the serum magnesium concentration and patient outcomes

Table 4 compares linear modelling (Model A) and two-segment nonlinear modelling (Model B) in relation to serum magnesium levels and mortality rates at 30 days, 60 days, and 90 days. The log-likelihood ratios for the probability of 30-day, 60-day, and 90-day mortality were 0.673, 0.224, and 0.221, respectively. All the *P* values were greater than 0.05. Based on these findings, it can be inferred that a linear model is more suitable for representing the relationship between serum magnesium and clinical outcomes.

**Table 4:** Linear and non-linear model of relationship between serum magnesium and outcomes

	Number(%)	OR(95%CI), *P*-value
**30-day mortality**		
Model A: The linear model	421	4.01 (1.22, 13.19) 0.022
Model B: Two-segment non-linear model		
The turning point of Serum magnesium(mg/dL)		
≤ 1.7(slope 1, left side)	212	8.10 (0.23, 283.95) 0.249
> 1.7(slope 2, right side)	209	3.12 (0.58, 16.65) 0.183
Slope 2 to slope 1		0.38 (0.00, 34.55) 0.677
Predicted at 1.7		-2.30 (-2.94, -1.66)
*P* for the log-likelihood ratio test		0.673
**60-day mortality**		
Model A: The linear model	421	4.81 (1.59, 14.53) 0.005
Model B: Two-segment non-linear model		
The turning point of Serum magnesium(mg/dL)		
≤1.8(slope 1, left side)	253	21.31 (1.33, 340.38) 0.031
>1.8(slope 2, right side)	168	2.14 (0.38, 12.03) 0.388
Slope 2 to slope 1		0.10 (0.00, 4.46) 0.235
Predicted at 1.8		-1.82 (-2.39, -1.26)
*P* for the log-likelihood ratio test		0.224
**90-day mortality**		
Model A: The linear model	421	4.45 (1.52, 12.96) 0.006
Model B: Two-segment non-linear model		
The turning point of Serum magnesium(mg/dL)		
≤1.8(slope 1, left side)	253	18.13 (1.35, 243.52) 0.029
>1.8(slope 2, right side)	168	2.00 (0.37, 10.78) 0.422
Slope 2 to slope 1		0.11 (0.00, 4.04) 0.230
Predicted at 1.8		-1.74 (-2.28, -1.19)
*P* for the log-likelihood ratio test		0.221

Model A is linear model, while Model B is two-segment non-linear. Both Model A and B were adjusted for age, gender, Hypertension, Diabetes, CAD, Renal disease, HR, DBP, SBP, RR, Calcium total, ALT, AST, BILIRUBINTOTAL, INR(s), PT(s), APTT(s), Creatinine, Urea nitrogen, Hematocrit, Hemoglobin, RBC, WBC, PLT, AG, Lactate, Glucose, APACHEII, SOFA. OR, odds ratio; CI, confidential interval

**Fig. 2:**
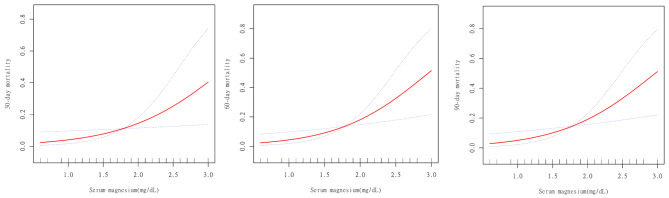
Smooth fitting curves demonstrating the linear association between serum magnesium levels and 30-day (**A**), 60-day (**B**), and 90-day (**C**) mortality

Figure 2 presents the smoothed fitting curve graph, which also illustrates a linear relationship between the serum magnesium concentration and clinical outcomes. From the graph, it was evident that as the serum magnesium concentration increased, the mortalities at 30 days, 60 days, and 90 days progressively increased.

### Kaplan–Meier analysis of mortality probabilities

**Fig. 3:**
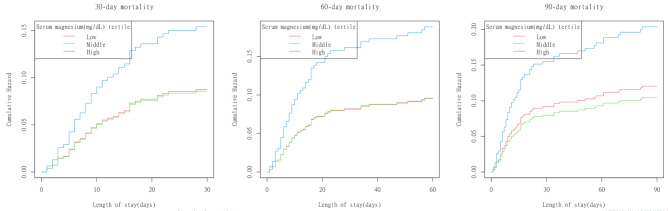
Kaplan–Meier analysis for the cumulative hazard of mortality at 30-day (**A**), 60-day (**B**) and 90-day (**C**) based on quartiles of serum magnesium levels from the Q1 to Q3 groups

Figure 3 shows that the hazard ratios of mortality in Group Q3 were significantly greater than those in Groups Q1 and Q2 at 30, 60, and 90 days. Additionally, the hazard ratios of mortality in Groups Q1, Q2, and Q3 increased with the prolongation of hospital stay, with a particularly noticeable trend in Group Q3.

## Discussion

In this study, we shed new light on the benefits of monitoring serum magnesium within 24 h of administration to predict the clinical outcome in septic patients with *E.coli* infection. There was a linear relationship between serum magnesium levels and clinical outcomes in E.coli septic patients. Every 1 mg/dL increase in serum magnesium led to obvious increases in 30-day, 60-day, and 90-day mortality rates of 301%, 381%, and 345%, respectively. To the best of our knowledge, this study is the first to investigate the relationship between serum magnesium and outcomes in septic patients with *E.coli* infection.

Magnesium is a vital ion within the human body and is involved in the physiological functions of the heart, brain, kidney, skeletal muscles, and intestine [21]. Magnesium also plays an important role in biochemical reactions and interacts with the body as a fundamental co-factor in various enzymatic reactions, modulating metabolism and protein synthesis while maintaining cell integrity [8]. Several studies have applied magnesium as a treatment for many critical diseases, such as preeclampsia, stroke, myocardial infarction, and asthma [22–24]. According to these basic and clinical studies, magnesium will never be considered a forgotten cation.

Emerging evidence has shed light on the relationship between magnesium and outcomes in critically ill patients. Niyada reported that serum magnesium concentrations greater than 2.4 mg/dL may inhibit cardiac function, as shown by the observed trend of QRS complex broadening, and serve as an independent marker for increased in-hospital mortality in patients admitted to the intensive cardiac care unit [25]. According to a single-centre cohort study, a discharge serum magnesium concentration ≥ 2.3 mg/dL is strongly linked to a greater risk for one-year mortality after discharge, regardless of the initial magnesium level at admission [26]. Previous reports on the relationship between magnesium and sepsis are controversial. Huabin’s findings indicate that hypermagnesemia, when other confounding variables such as acute kidney damage and disrupted calcium and potassium metabolism are excluded, is a significant predictor of death in children with sepsis [11]. However, a retrospective observational study reported that hypomagnesemia is associated with adverse clinical outcomes of sepsis, such as increased risk of disseminated intravascular coagulation and mortality [27]. The use of magnesium sulfate may decrease in-hospital mortality, ICU mortality and the use of renal replacement therapy [28].

*E.coli* is a strain of *gram*-negative commensal bacteria that colonizes the digestive tract of humans. Nevertheless, some of these bacteria are pathogenic and are responsible for severe bacterial infections [29]. As a result of the appearance of serotypes that are resistant to multiple antibiotics, *E.coli* is widely considered one of the most problematic bacteria for human infections, leading to enormous health care and social costs [30]. Although magnesium may be a potential predictor of outcomes in critically ill patients, as previously mentioned, few studies have investigated the relationships between serum magnesium and the risk of mortality in septic patients with *E.coli* infection. Our study indicated that serum magnesium is positively correlated with mortality at 30 days, 60 days, and 90 days after adjusting for all the potential confounding variables in septic patients with *E.coli* infection. Septic patients with serum magnesium levels greater than 1.9 mg/dL had greater mortality at 30 days, 60 days and 90 days.

Possible explanations for our findings are as follows. Magnesium assists in regulating enzymes involved in all metabolic pathways. It is essential for energy because ATP only has biological activity when combined with magnesium. Magnesium stabilizes macromolecules, regulates the activity of ion channels, and has also been reported to function as a second messenger in signal transduction [31]. Extracellular magnesium ions hinder the activity of voltage-gated Ca2 + channels in vascular smooth muscle cells, leading to a reduction in vasoconstriction and an increase in vasodilation [32]. When present within the normal physiological range, magnesium ions have been shown to increase the release of nitric oxide by endothelial cells, resulting in vascular vasodilation [33]. *E.coli* infection can release a large amount of endotoxins, leading to a decrease in vascular tension. In addition, magnesium affects the vascellum and induces much more severe hypotension. Furthermore, magnesium has a substantial inhibitory effect on cardiac potassium channels. These changes can occur in combination and result in malignant arrhythmias and heart failure [34]. It is worth noting that a two-component system PhoPQ has been proven to be crucial for the virulence of *E.coli*. This activated system, in turn, induces transcription of magnesium and increase resistance to antimicrobial peptides, thereby promoting *E.coli* survival [35]. Lactate can also function as a signaling molecule controlling magnesium processing between the endoplasmic reticulum and mitochondria. The bacterial endotoxin lipopolysaccharide enhances the lactate-mediated increase in magnesium, further enhancing mitochondrial reprogramming and multiple organ failure [36]. Elevated serum magnesium could be caused by a decrease in magnesium excretion through the kidney after acute kidney injury, which is a common complication of *E.coli* sepsis. In addition, serum magnesium levels may start to rise before the elevation of creatinine due to decreased urinary excretion of magnesium [37].

The strength of our study was that we conducted pioneering research investigating the correlation between the serum magnesium concentration and patient outcome in a population of septic patients with *E.coli* infection. Serum magnesium was associated with increased 30-day, 60-day and 90-day mortality. Serum magnesium is an easily obtained but overlooked indicator in clinical practice. Our study may help clinicians to understand the importance of this simple and convenient indicator and utilize this indicator for risk stratification and management in high-risk patients. In our study, some limitations should be noted. Firstly, several patients were excluded due to the absence of a serum magnesium indicator, and we didn’t evaluate how many patients have blood culture positive in sepsis. Thus, the study’s sample size was not large enough, making it critical to conduct studies with larger sample sizes in the future and providing more robust evidence. Secondly, study time was not enough to observe the relationship between serum magnesium and clinical outcomes in long-term. Thirdly, we did not obtain detailed pre-hospital information, background and intervening causes such as pre-hospital magnesium therapy are not considered, and the patient’s dialysis status may also affect the serum magnesium concentration. Fourthly, due to the lack of some data in the database, we didn’t analyze whether antimicrobial resistance in *E.coli* influence the association between magnesium levels and mortality, we will focus on these issue in the future research. Finally, Due to the nature of retrospective study, there is a possibility of missing some patient data that affects the statistical analysis.

## Conclusion

Increased level of serum magnesium is significantly associated with increased risk of 30-day, 60-day and 90-day mortality in a population of septic patients with *E.coli* infection. Therefore, serum magnesium might be a beneficial tool for risk stratification and management. Further investigations are required to validate these findings and contribute to understanding the underlying mechanisms involved.

## Data Availability

The data that support the findings of this study are available from the Massachusetts Institute of Technology (MIT) and Beth Israel Deaconess Medical Center (BIDMC) but restrictions apply to the availability of these data, which were used under license for the current study, and so are not publicly available. Data are however available from the authors upon reasonable request and with permission of the Massachusetts Institute of Technology (MIT) and Beth Israel Deaconess Medical Center (BIDMC).
